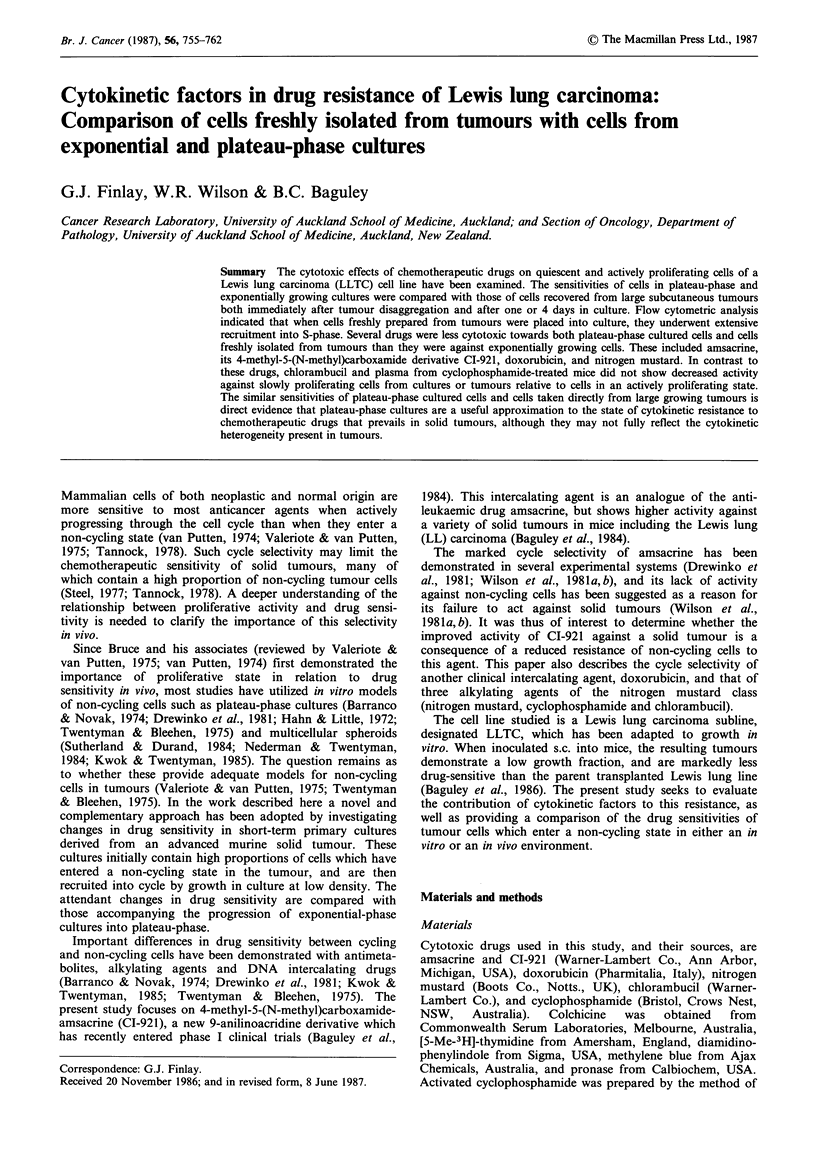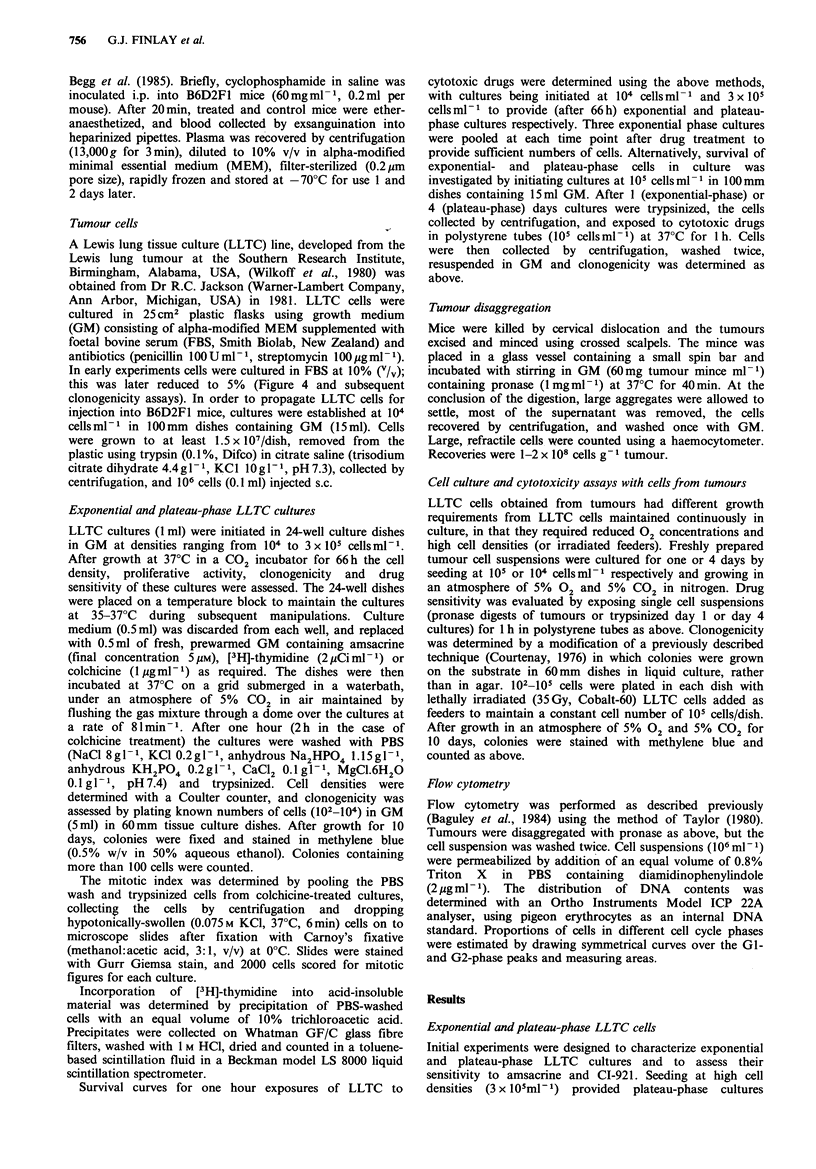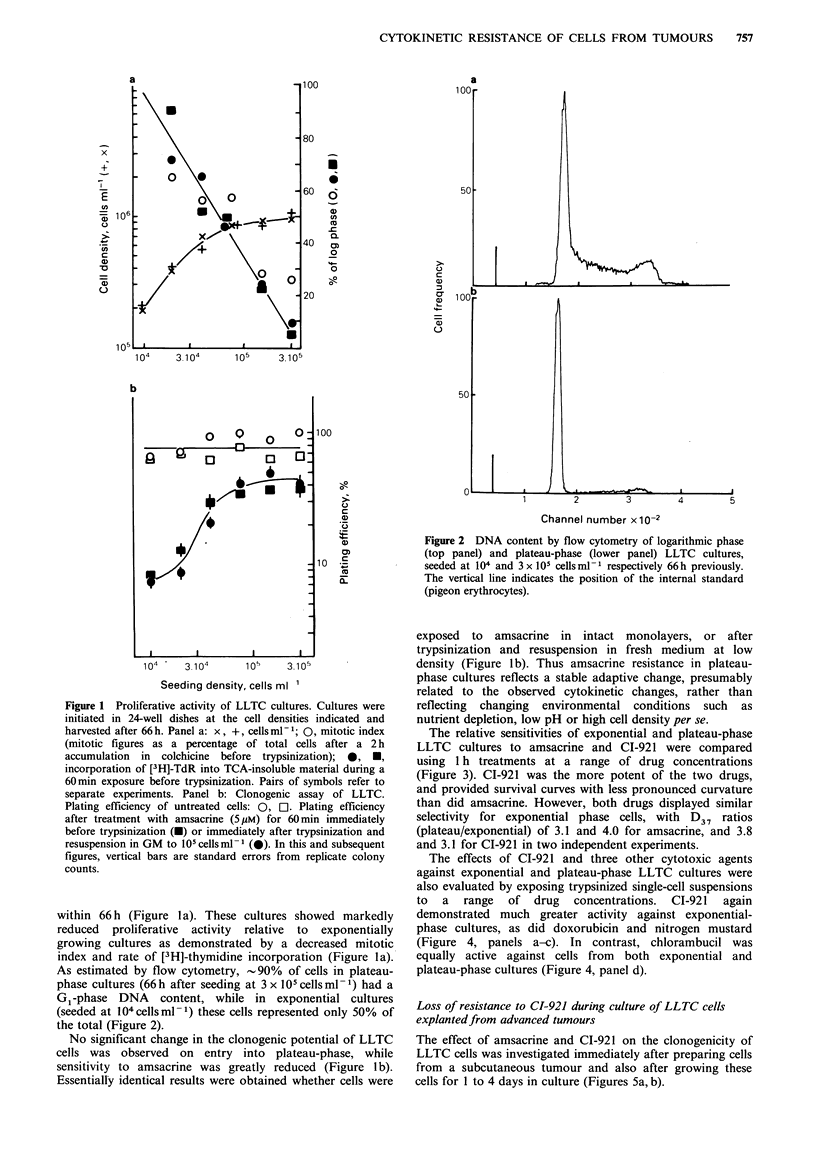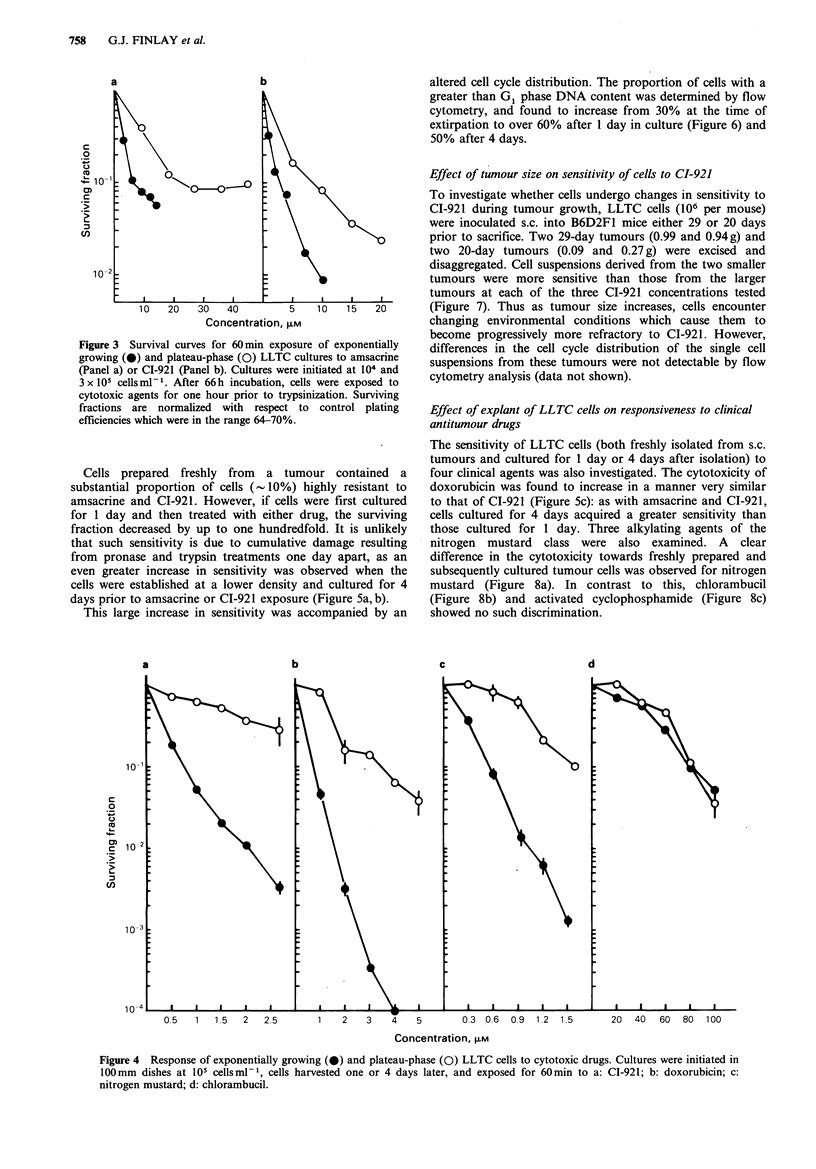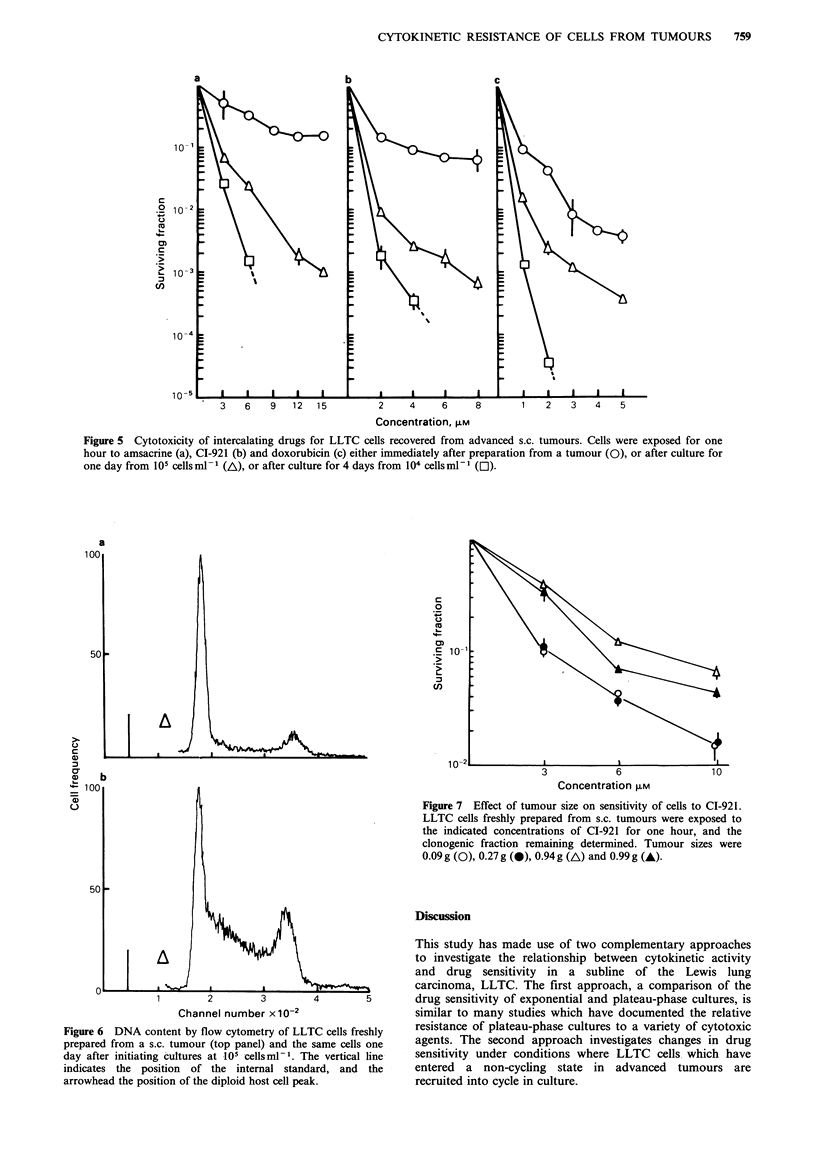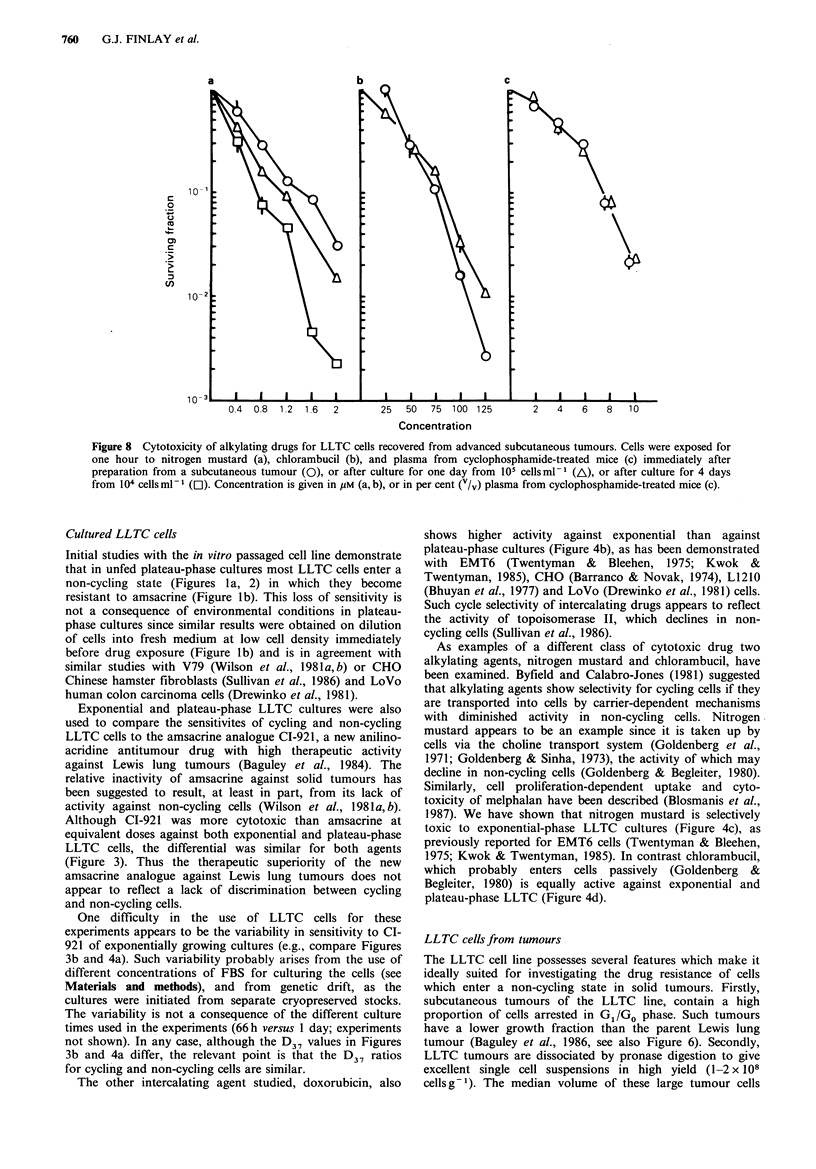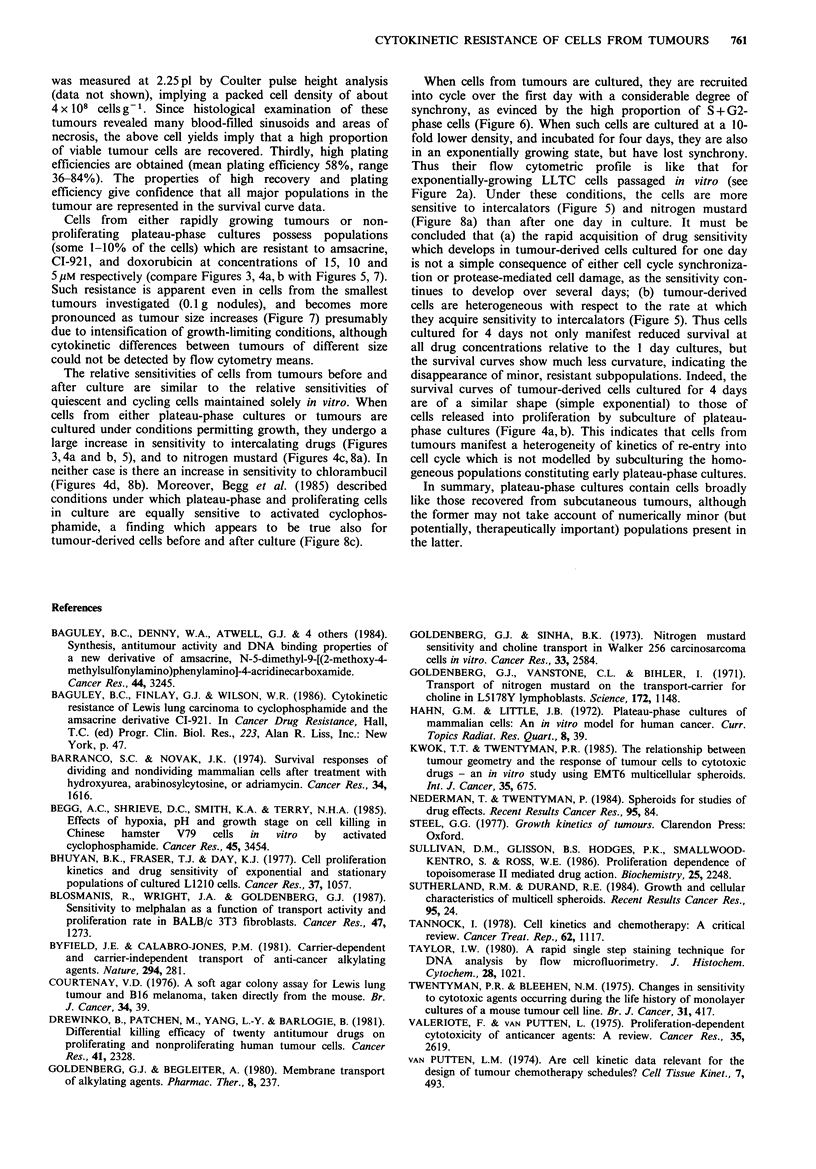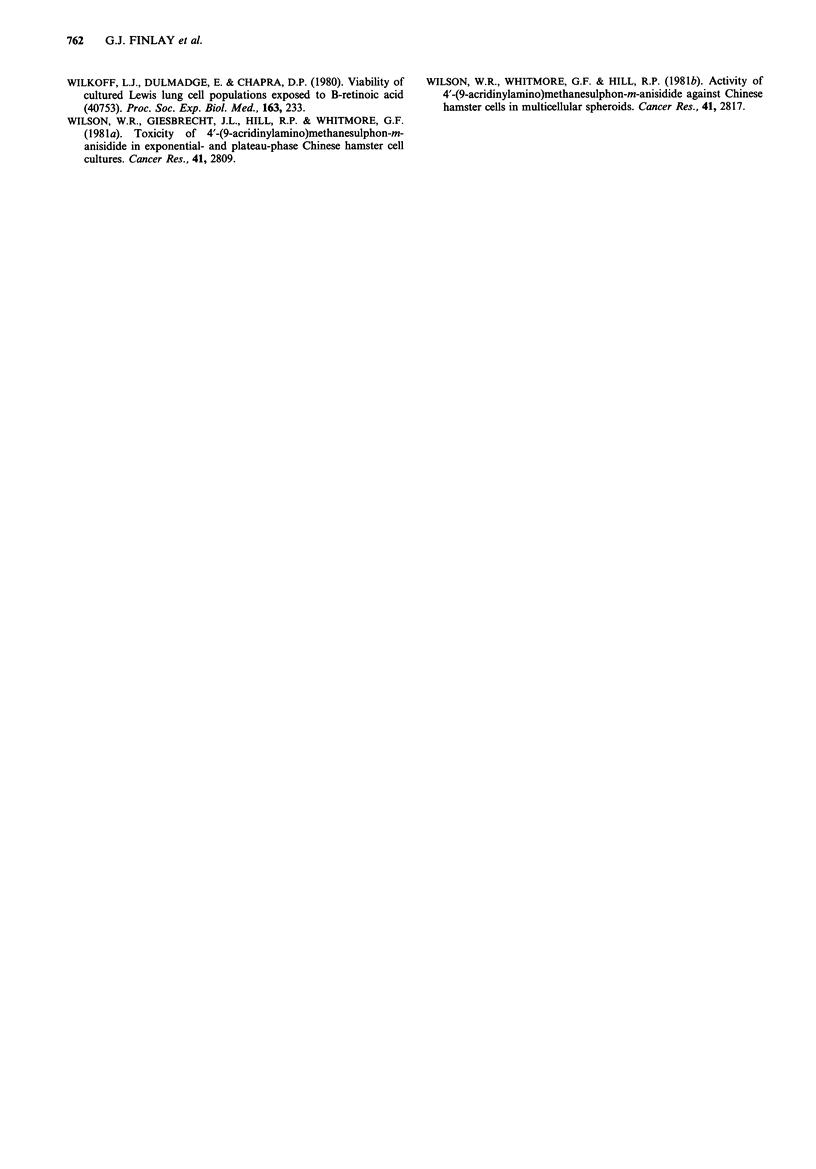# Cytokinetic factors in drug resistance of Lewis lung carcinoma: comparison of cells freshly isolated from tumours with cells from exponential and plateau-phase cultures.

**DOI:** 10.1038/bjc.1987.284

**Published:** 1987-12

**Authors:** G. J. Finlay, W. R. Wilson, B. C. Baguley

**Affiliations:** Cancer Research Laboratory, University of Auckland School of Medicine, New Zealand.

## Abstract

The cytotoxic effects of chemotherapeutic drugs on quiescent and actively proliferating cells of a Lewis lung carcinoma (LLTC) cell line have been examined. The sensitivities of cells in plateau-phase and exponentially growing cultures were compared with those of cells recovered from large subcutaneous tumours both immediately after tumour disaggregation and after one or 4 days in culture. Flow cytometric analysis indicated that when cells freshly prepared from tumours were placed into culture, they underwent extensive recruitment into S-phase. Several drugs were less cytotoxic towards both plateau-phase cultured cells and cells freshly isolated from tumours than they were against exponentially growing cells. These included amsacrine, its 4-methyl-5-(N-methyl)carboxamide derivative CI-921, doxorubicin, and nitrogen mustard. In contrast to these drugs, chlorambucil and plasma from cyclophosphamide-treated mice did not show decreased activity against slowly proliferating cells from cultures or tumours relative to cells in an actively proliferating state. The similar sensitivities of plateau-phase cultured cells and cells taken directly from large growing tumours is direct evidence that plateau-phase cultures are a useful approximation to the state of cytokinetic resistance to chemotherapeutic drugs that prevails in solid tumours, although they may not fully reflect the cytokinetic heterogeneity present in tumours.


*Br. J. Cancer* (1987), **56**, 755–762 © The Macmillan Press Ltd., 1987

### Cytokinetic factors in drug resistance of Lewis lung carcinoma: Comparison of cells freshly isolated from tumours with cells from exponential and plateau-phase cultures

G.J. Finlay, W.R. Wilson & B.C. Baguley

*Cancer Research Laboratory, University of Auckland School of Medicine, Auckland; and Section of Oncology, Department of Pathology, University of Auckland School of Medicine, Auckland, New Zealand.*

**Summary** The cytotoxic effects of chemotherapeutic drugs on quiescent and actively proliferating cells of a Lewis lung carcinoma (LLTC) cell line have been examined. The sensitivities of cells in plateau-phase and exponentially growing cultures were compared with those of cells recovered from large subcutaneous tumours both immediately after tumour disaggregation and after one or 4 days in culture. Flow cytometric analysis indicated that when cells freshly prepared from tumours were placed into culture, they underwent extensive recruitment into S-phase. Several drugs were less cytotoxic towards both plateau-phase cultured cells and cells freshly isolated from tumours than they were against exponentially growing cells. These included amsacrine, its 4-methyl-5-(N-methyl)carboxamide derivative CI-921, doxorubicin, and nitrogen mustard. In contrast to these drugs, chlorambucil and plasma from cyclophosphamide-treated mice did not show decreased activity against slowly proliferating cells from cultures or tumours relative to cells in an actively proliferating state. The similar sensitivities of plateau-phase cultured cells and cells taken directly from large growing tumours is direct evidence that plateau-phase cultures are a useful approximation to the state of cytokinetic resistance to chemotherapeutic drugs that prevails in solid tumours, although they may not fully reflect the cytokinetic heterogeneity present in tumours.

Mammalian cells of both neoplastic and normal origin are more sensitive to most anticancer agents when actively progressing through the cell cycle than when they enter a non-cycling state (van Putten, 1974; Valeriote & van Putten, 1975; Tannock, 1978). Such cycle selectivity may limit the chemotherapeutic sensitivity of solid tumours, many of which contain a high proportion of non-cycling tumour cells (Steel, 1977; Tannock, 1978). A deeper understanding of the relationship between proliferative activity and drug sensitivity is needed to clarify the importance of this selectivity *in vivo*.

Since Bruce and his associates (reviewed by Valeriote & van Putten, 1975; van Putten, 1974) first demonstrated the importance of proliferative state in relation to drug sensitivity *in vivo*, most studies have utilized *in vitro* models of non-cycling cells such as plateau-phase cultures (Barranco & Novak, 1974; Drewinko *et al.*, 1981; Hahn & Little, 1972; Twentyman & Bleehen, 1975) and multicellular spheroids (Sutherland & Durand, 1984; Nederman & Twentyman, 1984; Kwok & Twentyman, 1985). The question remains as to whether these provide adequate models for non-cycling cells in tumours (Valeriote & van Putten, 1975; Twentyman & Bleehen, 1975). In the work described here a novel and complementary approach has been adopted by investigating changes in drug sensitivity in short-term primary cultures derived from an advanced murine solid tumour. These cultures initially contain high proportions of cells which have entered a non-cycling state in the tumour, and are then recruited into cycle by growth in culture at low density. The attendant changes in drug sensitivity are compared with those accompanying the progression of exponential-phase cultures into plateau-phase.

Important differences in drug sensitivity between cycling and non-cycling cells have been demonstrated with antimetabolites, alkylating agents and DNA intercalating drugs (Barranco & Novak, 1974; Drewinko *et al.*, 1981; Kwok & Twentyman, 1985; Twentyman & Bleehen, 1975). The present study focuses on 4-methyl-5-(N-methyl)carboxamide-amsacrine (CI-921), a new 9-anilinoacridine derivative which has recently entered phase I clinical trials (Baguley *et al.*, 1984). This intercalating agent is an analogue of the anti-leukaemic drug amsacrine, but shows higher activity against a variety of solid tumours in mice including the Lewis lung (LL) carcinoma (Baguley *et al.*, 1984).

The marked cycle selectivity of amsacrine has been demonstrated in several experimental systems (Drewinko *et al.*, 1981; Wilson *et al.*, 1981*a,b*), and its lack of activity against non-cycling cells has been suggested as a reason for its failure to act against solid tumours (Wilson *et al.*, 1981*a,b*). It was thus of interest to determine whether the improved activity of CI-921 against a solid tumour is a consequence of a reduced resistance of non-cycling cells to this agent. This paper also describes the cycle selectivity of another clinical intercalating agent, doxorubicin, and that of three alkylating agents of the nitrogen mustard class (nitrogen mustard, cyclophosphamide and chlorambucil).

The cell line studied is a Lewis lung carcinoma subline, designated LLTC, which has been adapted to growth *in vitro*. When inoculated s.c. into mice, the resulting tumours demonstrate a low growth fraction, and are markedly less drug-sensitive than the parent transplanted Lewis lung line (Baguley *et al.*, 1986). The present study seeks to evaluate the contribution of cytokinetic factors to this resistance, as well as providing a comparison of the drug sensitivities of tumour cells which enter a non-cycling state in either an *in vitro* or an *in vivo* environment.

### Materials and methods

### *Materials*

Cytotoxic drugs used in this study, and their sources, are amsacrine and CI-921 (Warner-Lambert Co., Ann Arbor, Michigan, USA), doxorubicin (Pharmitalia, Italy), nitrogen mustard (Boots Co., Notts., UK), chlorambucil (Warner-Lambert Co.), and cyclophosphamide (Bristol, Crows Nest, NSW, Australia). Colchicine was obtained from Commonwealth Serum Laboratories, Melbourne, Australia, [5-Me-$^3$H]-thymidine from Amersham, England, diamidino-phenylindole from Sigma, USA, methylene blue from Ajax Chemicals, Australia, and pronase from Calbiochem, USA. Activated cyclophosphamide was prepared by the method of

Correspondence: G.J. Finlay.
Received 20 November 1986; and in revised form, 8 June 1987.

Begg *et al.* (1985). Briefly, cyclophosphamide in saline was inoculated i.p. into B6D2F1 mice ($60\,mg\,ml^{-1}$, 0.2 ml per mouse). After 20 min, treated and control mice were ether-anaesthetized, and blood collected by exsanguination into heparinized pipettes. Plasma was recovered by centrifugation (13,000 *g* for 3 min), diluted to 10% v/v in alpha-modified minimal essential medium (MEM), filter-sterilized ($0.2\,\mu m$ pore size), rapidly frozen and stored at $-70°C$ for use 1 and 2 days later.

### *Tumour cells*

A Lewis lung tissue culture (LLTC) line, developed from the Lewis lung tumour at the Southern Research Institute, Birmingham, Alabama, USA, (Wilkoff *et al.*, 1980) was obtained from Dr R.C. Jackson (Warner-Lambert Company, Ann Arbor, Michigan, USA) in 1981. LLTC cells were cultured in $25\,cm^2$ plastic flasks using growth medium (GM) consisting of alpha-modified MEM supplemented with foetal bovine serum (FBS, Smith Biolab, New Zealand) and antibiotics (penicillin $100\,U\,ml^{-1}$, streptomycin $100\,\mu g\,ml^{-1}$). In early experiments cells were cultured in FBS at 10% ($^v/_v$); this was later reduced to 5% (Figure 4 and subsequent clonogenicity assays). In order to propagate LLTC cells for injection into B6D2F1 mice, cultures were established at $10^4$ cells $ml^{-1}$ in 100 mm dishes containing GM (15 ml). Cells were grown to at least $1.5 \times 10^7$/dish, removed from the plastic using trypsin (0.1%, Difco) in citrate saline (trisodium citrate dihydrate $4.4\,g\,l^{-1}$, KCl $10\,g\,l^{-1}$, pH 7.3), collected by centrifugation, and $10^6$ cells (0.1 ml) injected s.c.

### *Exponential and plateau-phase LLTC cultures*

LLTC cultures (1 ml) were initiated in 24-well culture dishes in GM at densities ranging from $10^4$ to $3 \times 10^5$ cells $ml^{-1}$. After growth at 37°C in a $CO_2$ incubator for 66 h the cell density, proliferative activity, clonogenicity and drug sensitivity of these cultures were assessed. The 24-well dishes were placed on a temperature block to maintain the cultures at 35–37°C during subsequent manipulations. Culture medium (0.5 ml) was discarded from each well, and replaced with 0.5 ml of fresh, prewarmed GM containing amsacrine (final concentration $5\,\mu M$), [$^3$H]-thymidine ($2\,\mu Ci\,ml^{-1}$) or colchicine ($1\,\mu g\,ml^{-1}$) as required. The dishes were then incubated at 37°C on a grid submerged in a waterbath, under an atmosphere of 5% $CO_2$ in air maintained by flushing the gas mixture through a dome over the cultures at a rate of $8\,l\,min^{-1}$. After one hour (2 h in the case of colchicine treatment) the cultures were washed with PBS (NaCl $8\,g\,l^{-1}$, KCl $0.2\,g\,l^{-1}$, anhydrous $Na_2HPO_4$ $1.15\,g\,l^{-1}$, anhydrous $KH_2PO_4$ $0.2\,g\,l^{-1}$, $CaCl_2$ $0.1\,g\,l^{-1}$, $MgCl.6H_2O$ $0.1\,g\,l^{-1}$, pH 7.4) and trypsinized. Cell densities were determined with a Coulter counter, and clonogenicity was assessed by plating known numbers of cells ($10^2$–$10^4$) in GM (5 ml) in 60 mm tissue culture dishes. After growth for 10 days, colonies were fixed and stained in methylene blue (0.5% w/v in 50% aqueous ethanol). Colonies containing more than 100 cells were counted.

The mitotic index was determined by pooling the PBS wash and trypsinized cells from colchicine-treated cultures, collecting the cells by centrifugation and dropping hypotonically-swollen (0.075 M KCl, 37°C, 6 min) cells on to microscope slides after fixation with Carnoy's fixative (methanol:acetic acid, 3:1, v/v) at 0°C. Slides were stained with Gurr Giemsa stain, and 2000 cells scored for mitotic figures for each culture.

Incorporation of [$^3$H]-thymidine into acid-insoluble material was determined by precipitation of PBS-washed cells with an equal volume of 10% trichloroacetic acid. Precipitates were collected on Whatman GF/C glass fibre filters, washed with 1 M HCl, dried and counted in a toluene-based scintillation fluid in a Beckman model LS 8000 liquid scintillation spectrometer.

Survival curves for one hour exposures of LLTC to cytotoxic drugs were determined using the above methods, with cultures being initiated at $10^4$ cells $ml^{-1}$ and $3 \times 10^5$ cells $ml^{-1}$ to provide (after 66 h) exponential and plateau-phase cultures respectively. Three exponential phase cultures were pooled at each time point after drug treatment to provide sufficient numbers of cells. Alternatively, survival of exponential- and plateau-phase cells in culture was investigated by initiating cultures at $10^5$ cells $ml^{-1}$ in 100 mm dishes containing 15 ml GM. After 1 (exponential-phase) or 4 (plateau-phase) days cultures were trypsinized, the cells collected by centrifugation, and exposed to cytotoxic drugs in polystyrene tubes ($10^5$ cells $ml^{-1}$) at 37°C for 1 h. Cells were then collected by centrifugation, washed twice, resuspended in GM and clonogenicity was determined as above.

### *Tumour disaggregation*

Mice were killed by cervical dislocation and the tumours excised and minced using crossed scalpels. The mince was placed in a glass vessel containing a small spin bar and incubated with stirring in GM (60 mg tumour mince $ml^{-1}$) containing pronase ($1\,mg\,ml^{-1}$) at 37°C for 40 min. At the conclusion of the digestion, large aggregates were allowed to settle, most of the supernatant was removed, the cells recovered by centrifugation, and washed once with GM. Large, refractile cells were counted using a haemocytometer. Recoveries were $1$–$2 \times 10^8$ cells $g^{-1}$ tumour.

### *Cell culture and cytotoxicity assays with cells from tumours*

LLTC cells obtained from tumours had different growth requirements from LLTC cells maintained continuously in culture, in that they required reduced $O_2$ concentrations and high cell densities (or irradiated feeders). Freshly prepared tumour cell suspensions were cultured for one or 4 days by seeding at $10^5$ or $10^4$ cells $ml^{-1}$ respectively and growing in an atmosphere of 5% $O_2$ and 5% $CO_2$ in nitrogen. Drug sensitivity was evaluated by exposing single cell suspensions (pronase digests of tumours or trypsinized day 1 or day 4 cultures) for 1 h in polystyrene tubes as above. Clonogenicity was determined by a modification of a previously described technique (Courtenay, 1976) in which colonies were grown on the substrate in 60 mm dishes in liquid culture, rather than in agar. $10^2$–$10^5$ cells were plated in each dish with lethally irradiated (35 Gy, Cobalt-60) LLTC cells added as feeders to maintain a constant cell number of $10^5$ cells/dish. After growth in an atmosphere of 5% $O_2$ and 5% $CO_2$ for 10 days, colonies were stained with methylene blue and counted as above.

### *Flow cytometry*

Flow cytometry was performed as described previously (Baguley *et al.*, 1984) using the method of Taylor (1980). Tumours were disaggregated with pronase as above, but the cell suspension was washed twice. Cell suspensions ($10^6\,ml^{-1}$) were permeabilized by addition of an equal volume of 0.8% Triton X in PBS containing diamidinophenylindole ($2\,\mu g\,ml^{-1}$). The distribution of DNA contents was determined with an Ortho Instruments Model ICP 22A analyser, using pigeon erythrocytes as an internal DNA standard. Proportions of cells in different cell cycle phases were estimated by drawing symmetrical curves over the G1- and G2-phase peaks and measuring areas.

### Results

### *Exponential and plateau-phase LLTC cells*

Initial experiments were designed to characterize exponential and plateau-phase LLTC cultures and to assess their sensitivity to amsacrine and CI-921. Seeding at high cell densities ($3 \times 10^5 ml^{-1}$) provided plateau-phase cultures

Figure 1 Proliferative activity of LLTC cultures. Cultures were initiated in 24-well dishes at the cell densities indicated and harvested after 66 h. Panel a: ×, +, cells ml$^{-1}$; ○, mitotic index (mitotic figures as a percentage of total cells after a 2 h accumulation in colchicine before trypsinization); ●, ■, incorporation of [$^3$H]-TdR into TCA-insoluble material during a 60 min exposure before trypsinization. Pairs of symbols refer to separate experiments. Panel b: Clonogenic assay of LLTC. Plating efficiency of untreated cells: ○, □. Plating efficiency after treatment with amsacrine (5 $\mu$M) for 60 min immediately before trypsinization (■) or immediately after trypsinization and resuspension in GM to $10^5$ cells ml$^{-1}$ (●). In this and subsequent figures, vertical bars are standard errors from replicate colony counts.

Figure 2 DNA content by flow cytometry of logarithmic phase (top panel) and plateau-phase (lower panel) LLTC cultures, seeded at $10^4$ and $3 \times 10^5$ cells ml$^{-1}$ respectively 66 h previously. The vertical line indicates the position of the internal standard (pigeon erythrocytes).

within 66 h (Figure 1a). These cultures showed markedly reduced proliferative activity relative to exponentially growing cultures as demonstrated by a decreased mitotic index and rate of [$^3$H]-thymidine incorporation (Figure 1a). As estimated by flow cytometry, ~90% of cells in plateau-phase cultures (66 h after seeding at $3 \times 10^5$ cells ml$^{-1}$) had a $G_1$-phase DNA content, while in exponential cultures (seeded at $10^4$ cells ml$^{-1}$) these cells represented only 50% of the total (Figure 2).

No significant change in the clonogenic potential of LLTC cells was observed on entry into plateau-phase, while sensitivity to amsacrine was greatly reduced (Figure 1b). Essentially identical results were obtained whether cells were exposed to amsacrine in intact monolayers, or after trypsinization and resuspension in fresh medium at low density (Figure 1b). Thus amsacrine resistance in plateau-phase cultures reflects a stable adaptive change, presumably related to the observed cytokinetic changes, rather than reflecting changing environmental conditions such as nutrient depletion, low pH or high cell density *per se*.

The relative sensitivities of exponential and plateau-phase LLTC cultures to amsacrine and CI-921 were compared using 1 h treatments at a range of drug concentrations (Figure 3). CI-921 was the more potent of the two drugs, and provided survival curves with less pronounced curvature than did amsacrine. However, both drugs displayed similar selectivity for exponential phase cells, with $D_{37}$ ratios (plateau/exponential) of 3.1 and 4.0 for amsacrine, and 3.8 and 3.1 for CI-921 in two independent experiments.

The effects of CI-921 and three other cytotoxic agents against exponential and plateau-phase LLTC cultures were also evaluated by exposing trypsinized single-cell suspensions to a range of drug concentrations. CI-921 again demonstrated much greater activity against exponential-phase cultures, as did doxorubicin and nitrogen mustard (Figure 4, panels a–c). In contrast, chlorambucil was equally active against cells from both exponential and plateau-phase cultures (Figure 4, panel d).

### *Loss of resistance to CI-921 during culture of LLTC cells explanted from advanced tumours*

The effect of amsacrine and CI-921 on the clonogenicity of LLTC cells was investigated immediately after preparing cells from a subcutaneous tumour and also after growing these cells for 1 to 4 days in culture (Figures 5a, b).

**Figure 3** Survival curves for 60 min exposure of exponentially growing (●) and plateau-phase (○) LLTC cultures to amsacrine (Panel a) or CI-921 (Panel b). Cultures were initiated at $10^4$ and $3 \times 10^5$ cells $ml^{-1}$. After 66 h incubation, cells were exposed to cytotoxic agents for one hour prior to trypsinization. Surviving fractions are normalized with respect to control plating efficiencies which were in the range 64–70%.

Cells prepared freshly from a tumour contained a substantial proportion of cells (~10%) highly resistant to amsacrine and CI-921. However, if cells were first cultured for 1 day and then treated with either drug, the surviving fraction decreased by up to one hundredfold. It is unlikely that such sensitivity is due to cumulative damage resulting from pronase and trypsin treatments one day apart, as an even greater increase in sensitivity was observed when the cells were established at a lower density and cultured for 4 days prior to amsacrine or CI-921 exposure (Figure 5a, b).

This large increase in sensitivity was accompanied by an altered cell cycle distribution. The proportion of cells with a greater than $G_1$ phase DNA content was determined by flow cytometry, and found to increase from 30% at the time of extirpation to over 60% after 1 day in culture (Figure 6) and 50% after 4 days.

*Effect of tumour size on sensitivity of cells to CI-921*

To investigate whether cells undergo changes in sensitivity to CI-921 during tumour growth, LLTC cells ($10^6$ per mouse) were inoculated s.c. into B6D2F1 mice either 29 or 20 days prior to sacrifice. Two 29-day tumours (0.99 and 0.94 g) and two 20-day tumours (0.09 and 0.27 g) were excised and disaggregated. Cell suspensions derived from the two smaller tumours were more sensitive than those from the larger tumours at each of the three CI-921 concentrations tested (Figure 7). Thus as tumour size increases, cells encounter changing environmental conditions which cause them to become progressively more refractory to CI-921. However, differences in the cell cycle distribution of the single cell suspensions from these tumours were not detectable by flow cytometry analysis (data not shown).

*Effect of explant of LLTC cells on responsiveness to clinical antitumour drugs*

The sensitivity of LLTC cells (both freshly isolated from s.c. tumours and cultured for 1 day or 4 days after isolation) to four clinical agents was also investigated. The cytotoxicity of doxorubicin was found to increase in a manner very similar to that of CI-921 (Figure 5c): as with amsacrine and CI-921, cells cultured for 4 days acquired a greater sensitivity than those cultured for 1 day. Three alkylating agents of the nitrogen mustard class were also examined. A clear difference in the cytotoxicity towards freshly prepared and subsequently cultured tumour cells was observed for nitrogen mustard (Figure 8a). In contrast to this, chlorambucil (Figure 8b) and activated cyclophosphamide (Figure 8c) showed no such discrimination.

**Figure 4** Response of exponentially growing (●) and plateau-phase (○) LLTC cells to cytotoxic drugs. Cultures were initiated in 100 mm dishes at $10^5$ cells $ml^{-1}$, cells harvested one or 4 days later, and exposed for 60 min to a: CI-921; b: doxorubicin; c: nitrogen mustard; d: chlorambucil.

**Figure 5** Cytotoxicity of intercalating drugs for LLTC cells recovered from advanced s.c. tumours. Cells were exposed for one hour to amsacrine (a), CI-921 (b) and doxorubicin (c) either immediately after preparation from a tumour (○), or after culture for one day from $10^5$ cells $ml^{-1}$ (△), or after culture for 4 days from $10^4$ cells $ml^{-1}$ (□).

**Figure 6** DNA content by flow cytometry of LLTC cells freshly prepared from a s.c. tumour (top panel) and the same cells one day after initiating cultures at $10^5$ cells $ml^{-1}$. The vertical line indicates the position of the internal standard, and the arrowhead the position of the diploid host cell peak.

**Figure 7** Effect of tumour size on sensitivity of cells to CI-921. LLTC cells freshly prepared from s.c. tumours were exposed to the indicated concentrations of CI-921 for one hour, and the clonogenic fraction remaining determined. Tumour sizes were 0.09 g (○), 0.27 g (●), 0.94 g (△) and 0.99 g (▲).

### Discussion

This study has made use of two complementary approaches to investigate the relationship between cytokinetic activity and drug sensitivity in a subline of the Lewis lung carcinoma, LLTC. The first approach, a comparison of the drug sensitivity of exponential and plateau-phase cultures, is similar to many studies which have documented the relative resistance of plateau-phase cultures to a variety of cytotoxic agents. The second approach investigates changes in drug sensitivity under conditions where LLTC cells which have entered a non-cycling state in advanced tumours are recruited into cycle in culture.

**Figure 8** Cytotoxicity of alkylating drugs for LLTC cells recovered from advanced subcutaneous tumours. Cells were exposed for one hour to nitrogen mustard (a), chlorambucil (b), and plasma from cyclophosphamide-treated mice (c) immediately after preparation from a subcutaneous tumour (○), or after culture for one day from $10^5$ cells $ml^{-1}$ (△), or after culture for 4 days from $10^4$ cells $ml^{-1}$ (□). Concentration is given in μM (a, b), or in per cent ($^V/_V$) plasma from cyclophosphamide-treated mice (c).

*Cultured LLTC cells*

Initial studies with the *in vitro* passaged cell line demonstrate that in unfed plateau-phase cultures most LLTC cells enter a non-cycling state (Figures 1a, 2) in which they become resistant to amsacrine (Figure 1b). This loss of sensitivity is not a consequence of environmental conditions in plateau-phase cultures since similar results were obtained on dilution of cells into fresh medium at low cell density immediately before drug exposure (Figure 1b) and is in agreement with similar studies with V79 (Wilson *et al.*, 1981*a,b*) or CHO Chinese hamster fibroblasts (Sullivan *et al.*, 1986) and LoVo human colon carcinoma cells (Drewinko *et al.*, 1981).

Exponential and plateau-phase LLTC cultures were also used to compare the sensitivites of cycling and non-cycling LLTC cells to the amsacrine analogue CI-921, a new anilino-acridine antitumour drug with high therapeutic activity against Lewis lung tumours (Baguley *et al.*, 1984). The relative inactivity of amsacrine against solid tumours has been suggested to result, at least in part, from its lack of activity against non-cycling cells (Wilson *et al.*, 1981*a,b*). Although CI-921 was more cytotoxic than amsacrine at equivalent doses against both exponential and plateau-phase LLTC cells, the differential was similar for both agents (Figure 3). Thus the therapeutic superiority of the new amsacrine analogue against Lewis lung tumours does not appear to reflect a lack of discrimination between cycling and non-cycling cells.

One difficulty in the use of LLTC cells for these experiments appears to be the variability in sensitivity to CI-921 of exponentially growing cultures (e.g., compare Figures 3b and 4a). Such variability probably arises from the use of different concentrations of FBS for culturing the cells (see **Materials and methods**), and from genetic drift, as the cultures were initiated from separate cryopreserved stocks. The variability is not a consequence of the different culture times used in the experiments (66 h *versus* 1 day; experiments not shown). In any case, although the $D_{37}$ values in Figures 3b and 4a differ, the relevant point is that the $D_{37}$ ratios for cycling and non-cycling cells are similar.

The other intercalating agent studied, doxorubicin, also shows higher activity against exponential than against plateau-phase cultures (Figure 4b), as has been demonstrated with EMT6 (Twentyman & Bleehen, 1975; Kwok & Twentyman, 1985), CHO (Barranco & Novak, 1974), L1210 (Bhuyan *et al.*, 1977) and LoVo (Drewinko *et al.*, 1981) cells. Such cycle selectivity of intercalating drugs appears to reflect the activity of topoisomerase II, which declines in non-cycling cells (Sullivan *et al.*, 1986).

As examples of a different class of cytotoxic drug two alkylating agents, nitrogen mustard and chlorambucil, have been examined. Byfield and Calabro-Jones (1981) suggested that alkylating agents show selectivity for cycling cells if they are transported into cells by carrier-dependent mechanisms with diminished activity in non-cycling cells. Nitrogen mustard appears to be an example since it is taken up by cells via the choline transport system (Goldenberg *et al.*, 1971; Goldenberg & Sinha, 1973), the activity of which may decline in non-cycling cells (Goldenberg & Begleiter, 1980). Similarly, cell proliferation-dependent uptake and cytotoxicity of melphalan have been described (Blosmanis *et al.*, 1987). We have shown that nitrogen mustard is selectively toxic to exponential-phase LLTC cultures (Figure 4c), as previously reported for EMT6 cells (Twentyman & Bleehen, 1975; Kwok & Twentyman, 1985). In contrast chlorambucil, which probably enters cells passively (Goldenberg & Begleiter, 1980) is equally active against exponential and plateau-phase LLTC (Figure 4d).

*LLTC cells from tumours*

The LLTC cell line possesses several features which make it ideally suited for investigating the drug resistance of cells which enter a non-cycling state in solid tumours. Firstly, subcutaneous tumours of the LLTC line, contain a high proportion of cells arrested in $G_1/G_0$ phase. Such tumours have a lower growth fraction than the parent Lewis lung tumour (Baguley *et al.*, 1986, see also Figure 6). Secondly, LLTC tumours are dissociated by pronase digestion to give excellent single cell suspensions in high yield ($1–2 \times 10^8$ cells $g^{-1}$). The median volume of these large tumour cells was measured at 2.25 pl by Coulter pulse height analysis (data not shown), implying a packed cell density of about $4 \times 10^8$ cells $g^{-1}$. Since histological examination of these tumours revealed many blood-filled sinusoids and areas of necrosis, the above cell yields imply that a high proportion of viable tumour cells are recovered. Thirdly, high plating efficiencies are obtained (mean plating efficiency 58%, range 36–84%). The properties of high recovery and plating efficiency give confidence that all major populations in the tumour are represented in the survival curve data.

Cells from either rapidly growing tumours or non-proliferating plateau-phase cultures possess populations (some 1–10% of the cells) which are resistant to amsacrine, CI-921, and doxorubicin at concentrations of 15, 10 and 5 $\mu$M respectively (compare Figures 3, 4a, b with Figures 5, 7). Such resistance is apparent even in cells from the smallest tumours investigated (0.1 g nodules), and becomes more pronounced as tumour size increases (Figure 7) presumably due to intensification of growth-limiting conditions, although cytokinetic differences between tumours of different size could not be detected by flow cytometry means.

The relative sensitivities of cells from tumours before and after culture are similar to the relative sensitivities of quiescent and cycling cells maintained solely *in vitro*. When cells from either plateau-phase cultures or tumours are cultured under conditions permitting growth, they undergo a large increase in sensitivity to intercalating drugs (Figures 3, 4a and b, 5), and to nitrogen mustard (Figures 4c, 8a). In neither case is there an increase in sensitivity to chlorambucil (Figures 4d, 8b). Moreover, Begg *et al.* (1985) described conditions under which plateau-phase and proliferating cells in culture are equally sensitive to activated cyclophosphamide, a finding which appears to be true also for tumour-derived cells before and after culture (Figure 8c).

When cells from tumours are cultured, they are recruited into cycle over the first day with a considerable degree of synchrony, as evinced by the high proportion of S+G2-phase cells (Figure 6). When such cells are cultured at a 10-fold lower density, and incubated for four days, they are also in an exponentially growing state, but have lost synchrony. Thus their flow cytometric profile is like that for exponentially-growing LLTC cells passaged *in vitro* (see Figure 2a). Under these conditions, the cells are more sensitive to intercalators (Figure 5) and nitrogen mustard (Figure 8a) than after one day in culture. It must be concluded that (a) the rapid acquisition of drug sensitivity which develops in tumour-derived cells cultured for one day is not a simple consequence of either cell cycle synchronization or protease-mediated cell damage, as the sensitivity continues to develop over several days; (b) tumour-derived cells are heterogeneous with respect to the rate at which they acquire sensitivity to intercalators (Figure 5). Thus cells cultured for 4 days not only manifest reduced survival at all drug concentrations relative to the 1 day cultures, but the survival curves show much less curvature, indicating the disappearance of minor, resistant subpopulations. Indeed, the survival curves of tumour-derived cells cultured for 4 days are of a similar shape (simple exponential) to those of cells released into proliferation by subculture of plateau-phase cultures (Figure 4a, b). This indicates that cells from tumours manifest a heterogeneity of kinetics of re-entry into cell cycle which is not modelled by subculturing the homogeneous populations constituting early plateau-phase cultures.

In summary, plateau-phase cultures contain cells broadly like those recovered from subcutaneous tumours, although the former may not take account of numerically minor (but potentially, therapeutically important) populations present in the latter.

### References

BAGULEY, B.C., DENNY, W.A., ATWELL, G.J. & 4 others (1984). Synthesis, antitumour activity and DNA binding properties of a new derivative of amsacrine, N-5-dimethyl-9-[(2-methoxy-4-methylsulfonylamino)phenylamino]-4-acridinecarboxamide. *Cancer Res.*, **44**, 3245.

BAGULEY, B.C., FINLAY, G.J. & WILSON, W.R. (1986). Cytokinetic resistance of Lewis lung carcinoma to cyclophosphamide and the amsacrine derivative CI-921. In *Cancer Drug Resistance*, Hall, T.C. (ed) Progr. Clin. Biol. Res., *223*, Alan R. Liss, Inc.: New York, p. 47.

BARRANCO, S.C. & NOVAK, J.K. (1974). Survival responses of dividing and nondividing mammalian cells after treatment with hydroxyurea, arabinosylcytosine, or adriamycin. *Cancer Res.*, **34**, 1616.

BEGG, A.C., SHRIEVE, D.C., SMITH, K.A. & TERRY, N.H.A. (1985). Effects of hypoxia, pH and growth stage on cell killing in Chinese hamster V79 cells *in vitro* by activated cyclophosphamide. *Cancer Res.*, **45**, 3454.

BHUYAN, B.K., FRASER, T.J. & DAY, K.J. (1977). Cell proliferation kinetics and drug sensitivity of exponential and stationary populations of cultured L1210 cells. *Cancer Res.*, **37**, 1057.

BLOSMANIS, R., WRIGHT, J.A. & GOLDENBERG, G.J. (1987). Sensitivity to melphalan as a function of transport activity and proliferation rate in BALB/c 3T3 fibroblasts. *Cancer Res.*, **47**, 1273.

BYFIELD, J.E. & CALABRO-JONES, P.M. (1981). Carrier-dependent and carrier-independent transport of anti-cancer alkylating agents. *Nature*, **294**, 281.

COURTENAY, V.D. (1976). A soft agar colony assay for Lewis lung tumour and B16 melanoma, taken directly from the mouse. *Br. J. Cancer*, **34**, 39.

DREWINKO, B., PATCHEN, M., YANG, L.-Y. & BARLOGIE, B. (1981). Differential killing efficacy of twenty antitumour drugs on proliferating and nonproliferating human tumour cells. *Cancer Res.*, **41**, 2328.

GOLDENBERG, G.J. & BEGLEITER, A. (1980). Membrane transport of alkylating agents. *Pharmac. Ther.*, **8**, 237.

GOLDENBERG, G.J. & SINHA, B.K. (1973). Nitrogen mustard sensitivity and choline transport in Walker 256 carcinosarcoma cells *in vitro*. *Cancer Res.*, **33**, 2584.

GOLDENBERG, G.J., VANSTONE, C.L. & BIHLER, I. (1971). Transport of nitrogen mustard on the transport-carrier for choline in L5178Y lymphoblasts. *Science*, **172**, 1148.

HAHN, G.M. & LITTLE, J.B. (1972). Plateau-phase cultures of mammalian cells: An *in vitro* model for human cancer. *Curr. Topics Radiat. Res. Quart.*, **8**, 39.

KWOK, T.T. & TWENTYMAN, P.R. (1985). The relationship between tumour geometry and the response of tumour cells to cytotoxic drugs – an *in vitro* study using EMT6 multicellular spheroids. *Int. J. Cancer*, **35**, 675.

NEDERMAN, T. & TWENTYMAN, P. (1984). Spheroids for studies of drug effects. *Recent Results Cancer Res.*, **95**, 84.

STEEL, G.G. (1977). *Growth kinetics of tumours*. Clarendon Press: Oxford.

SULLIVAN, D.M., GLISSON, B.S. HODGES, P.K., SMALLWOOD-KENTRO, S. & ROSS, W.E. (1986). Proliferation dependence of topoisomerase II mediated drug action. *Biochemistry*, **25**, 2248.

SUTHERLAND, R.M. & DURAND, R.E. (1984). Growth and cellular characteristics of multicell spheroids. *Recent Results Cancer Res.*, **95**, 24.

TANNOCK, I. (1978). Cell kinetics and chemotherapy: A critical review. *Cancer Treat. Rep.*, **62**, 1117.

TAYLOR, I.W. (1980). A rapid single step staining technique for DNA analysis by flow microfluorimetry. *J. Histochem. Cytochem.*, **28**, 1021.

TWENTYMAN, P.R. & BLEEHEN, N.M. (1975). Changes in sensitivity to cytotoxic agents occurring during the life history of monolayer cultures of a mouse tumour cell line. *Br. J. Cancer*, **31**, 417.

VALERIOTE, F. & VAN PUTTEN, L. (1975). Proliferation-dependent cytotoxicity of anticancer agents: A review. *Cancer Res.*, **35**, 2619.

VAN PUTTEN, L.M. (1974). Are cell kinetic data relevant for the design of tumour chemotherapy schedules? *Cell Tissue Kinet.*, **7**, 493.

WILKOFF, L.J., DULMADGE, E. & CHAPRA, D.P. (1980). Viability of cultured Lewis lung cell populations exposed to B-retinoic acid (40753). *Proc. Soc. Exp. Biol. Med.*, **163**, 233.

WILSON, W.R., GIESBRECHT, J.L., HILL, R.P. & WHITMORE, G.F. (1981*a*). Toxicity of 4′-(9-acridinylamino)methanesulphon-*m*-anisidide in exponential- and plateau-phase Chinese hamster cell cultures. *Cancer Res.*, **41**, 2809.

WILSON, W.R., WHITMORE, G.F. & HILL, R.P. (1981*b*). Activity of 4′-(9-acridinylamino)methanesulphon-*m*-anisidide against Chinese hamster cells in multicellular spheroids. *Cancer Res.*, **41**, 2817.